# Protein kinase D1 (PKD1) activation mediates a compensatory protective response during early stages of oxidative stress-induced neuronal degeneration

**DOI:** 10.1186/1750-1326-6-43

**Published:** 2011-06-22

**Authors:** Arunkumar Asaithambi, Arthi Kanthasamy, Hariharan Saminathan, Vellareddy Anantharam, Anumantha G Kanthasamy

**Affiliations:** 1 Department of Biomedical Sciences, 2062 Veterinary Medicine Bldg, Iowa State University, Ames, IA 50011, USA

## Abstract

**Background:**

Oxidative stress is a key pathophysiological mechanism contributing to degenerative processes in many neurodegenerative diseases and therefore, unraveling molecular mechanisms underlying various stages of oxidative neuronal damage is critical to better understanding the diseases and developing new treatment modalities. We previously showed that protein kinase C delta (PKCδ) proteolytic activation during the late stages of oxidative stress is a key proapoptotic signaling mechanism that contributes to oxidative damage in Parkinson's disease (PD) models. The time course studies revealed that PKCδ activation precedes apoptotic cell death and that cells resisted early insults of oxidative damage, suggesting that some intrinsic compensatory response protects neurons from early oxidative insult. Therefore, the purpose of the present study was to characterize protective signaling pathways in dopaminergic neurons during early stages of oxidative stress.

**Results:**

Herein, we identify that protein kinase D1 (PKD1) functions as a key anti-apoptotic kinase to protect neuronal cells against early stages of oxidative stress. Exposure of dopaminergic neuronal cells to H_2_O_2 _or 6-OHDA induced PKD1 activation loop (PKD1S744/748) phosphorylation long before induction of neuronal cell death. Blockade of PKCδ cleavage, PKCδ knockdown or overexpression of a cleavage-resistant PKCδ mutant effectively attenuated PKD1 activation, indicating that PKCδ proteolytic activation regulates PKD1 phosphorylation. Furthermore, the PKCδ catalytic fragment, but not the regulatory fragment, increased PKD1 activation, confirming PKCδ activity modulates PKD1 activation. We also identified that phosphorylation of S916 at the C-terminal is a preceding event required for PKD1 activation loop phosphorylation. Importantly, negative modulation of PKD1 by the RNAi knockdown or overexpression of PKD1^S916A ^phospho-defective mutants augmented oxidative stress-induced apoptosis, while positive modulation of PKD1 by the overexpression of full length PKD1 or constitutively active PKD1 plasmids attenuated oxidative stress-induced apoptosis, suggesting an anti-apoptotic role for PKD1 during oxidative neuronal injury.

**Conclusion:**

Collectively, our results demonstrate that PKCδ-dependent activation of PKD1 represents a novel intrinsic protective response in counteracting early stage oxidative damage in neuronal cells. Our results suggest that positive modulation of the PKD1-mediated compensatory protective mechanism against oxidative damage in dopaminergic neurons may provide novel neuroprotective strategies for treatment of PD.

## Background

Oxidative stress-induced neuronal damage has been implicated in many neurodegenerative disorders including Parkinson's disease (PD), Alzheimer's diseases, ALS, Huntington's diseases and stroke [1-7]. Neuronal cells maintain an oxidant/antioxidant homeostatic balance, and any breach in redox homeostasis causes excessive ROS production, resulting in oxidative damage [8-10]. Oxidative stress triggers apoptosis through activation of many signaling molecules including kinases and proteases [11-15], and the roles of these signaling molecules in dopaminergic cell death are being actively investigated. Recently, we demonstrated that the proteolytic activation of PKCδ, a novel PKC family member, mediates apoptosis in cell culture and animal models of PD [15-19].

PKCδ can be activated by membrane translocation, phosphorylation, or proteolytic cleavage by caspase-3, leading to persistently active catalytic fragments. We previously showed that various oxidative stressors like H_2_O_2_, MPP^+ ^and 6-OHDA induce PKCδ cleavage to increase the kinase activity and apoptosis in dopaminergic cells [20,15,21,16]. The time course studies revealed that the pro-apoptotic proteolytic activation of PKCδ occurs well before apoptotic cell death, and that cells resist early oxidative damage, suggesting that some key intrinsic compensatory responses protect neurons from the initial oxidative insult. Therefore, we speculated that the persistently active catalytic fragment of PKCδ might have other functions during the early stages of oxidative stress, and so we further explored downstream signaling mechanisms.

Protein kinase D1 (PKD1) is a calcium/calmodulin-dependent member of the CAMK kinase family and can be activated by dual phosphorylation of seine residues (Ser 744/748) in the catalytic domain by different PKCs, depending upon the cellular type and stimuli [22-24]. PKD1 is activated in response to multiple stimuli including growth factors, phorbol esters, G-protein coupled receptors, genotoxic stress and oxidative stress [25-28]. In non-neuronal cells, PKD1 activation has been shown to play a role in diverse cellular functions including proliferation, cytoskeletal reorganization, golgi function and immune response [27,29-32]. PKD1 has been shown to regulate various cell signaling molecules and pathways including ERK1/2, JNK pathways [33-35], effector enzymes like MnSOD that scavenge ROS [31,36], transcriptional regulators including NF-κB and MEF2 [37,38], stress responsive chaperones like HSP27 [39], and some members of the HDAC family [26,31,40]. Recently, PKD1 was recognized as an important mitochondrial ROS sensor that translocates to the nucleus to switch on cell survival mechanisms [36]. Also, PKD1 activation loop phosphorylation has been shown as an early marker of sympathetic neuronal DNA damage [41]. In neuronal models, PKD1 regulates trafficking of dendritic membrane proteins to maintain neuronal polarity and synaptic plasticity [42].

While many biological functions of PKD1 are beginning to emerge, the role of PKD1 in the brain, specifically in the nigral dopaminergic system, remains unknown. The relationship between PKD1 signaling and neurodegeneration has not yet been examined in a single study. Herein, we demonstrate that PKD1 closely interacts with PKCδ and serves as a key compensatory protective mechanism in dopaminergic neuronal cells during the early stages of oxidative insult.

## Results

### Role of PKCδ cleavage in the early stages of H_2_O_2_-induced oxidative stress in dopaminergic neuronal cells

**Figure 1:**
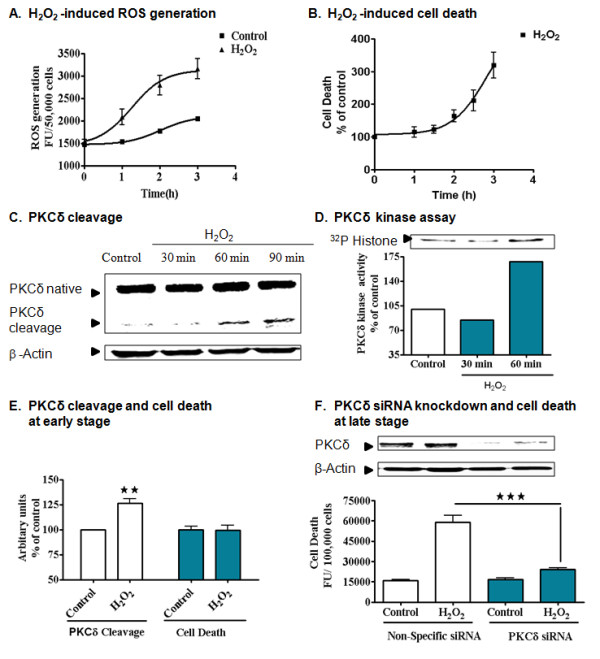
**Relationship between PKCδ proteolytic activation and cell death during initial stages of H_2_O_2 _-induced oxidative stress in N27 dopaminergic neuronal cell model**. N27 dopaminergic cells were treated with H_2_O_2 _(100 μM) for 0-180 min and assayed for ROS generation using DCFDA dye (A) and for cytotoxicity using Sytox green dye (B). Non-linear regression graph from two or more independent experiments (n = 6-8). PKCδ cleavage was monitored by Western blot in a time dependent manner for 0 - 90 min in N27 dopaminergic cells treated with H_2_O_2 _(100 μM) (C). N27 dopaminergic cells were treated with H_2_O_2 _(100 μM) for 0, 30 or 60 minutes and PKCδ kinase activity was measured using [^32^P] kinase assay; the bands were quantified for the graph (D). Data quantified from B and C were used to generate a bar graph and were compared with cytotoxicity and PKCδ cleavage following H_2_O_2 _exposure for 60 min. **, p < 0.01 as indicated by two-way ANOVA analysis using Bonferroni post test (E). N27 cells were transfected with 1 μM PKCδ siRNA and non-specific siRNA for 24 h and treated with 100 μM H_2_O_2 _and monitored for cytotoxicity using Sytox green dye, which showed significant protection from oxidative stress. ***, p < 0.001 denotes significant difference between non-specific siRNA- H_2_O_2 _and PKCδ siRNA H_2_O_2_-treated groups from two or more independent experiments (n = 6-8). Statistics were performed by one-way ANOVA analysis using Bonferroni post test (F).

H_2_O_2 _is a common oxidative insult used to probe various cellular signaling pathways in different cell types including neuronal cells [15,43,44]. We have already demonstrated that H_2_O_2 _causes dose- and time-dependent cytotoxicity, DNA fragmentation and cell death in the dopaminergic neuronal N27 cell model [15]. In order to determine the interrelationship between PKCδ proteolytic cleavage and oxidative stress-induced cell death, we examined the time course of PKCδ proteolytic activation and cell death. As shown in Figure 1A, generation of ROS occurs as early as 1 h after H_2_O_2 _treatment. Cytotoxicity begins between 90 and 120 min (Figure 1B). PKCδ proteolytic cleavage and the kinase activity increases during the early stage of H_2_O_2 _exposure at 60 min (Figure 1C, D). Comparison of PKCδ proteolytic activation and cytotoxicity at 60 min revealed that PKCδ proteolytic activation occurs during the early stages of oxidative stress preceding cell death (Figure 1E). There was no cell death during this intermediate period between ROS generation and PKCδ proteolytic activation. PKCδ knockdown by RNAi almost completely blocked the cell death induced by H_2_O_2 _at 120 min of treatment, demonstrating the pro-apoptotic function performed by PKCδ in N27 cells (Figure 1F). Importantly, a significant lag period between induction of cell death and PKCδ proteolytic activation was observed. This interesting observation prompted a search for cell signaling mechanisms associated with a compensatory protective response involving PKCδ proteolytic activation during early stages of oxidative stress.

### Oxidative stress induces phosphorylation and activation of PKD1 in a time-dependent manner

**Figure 2:**
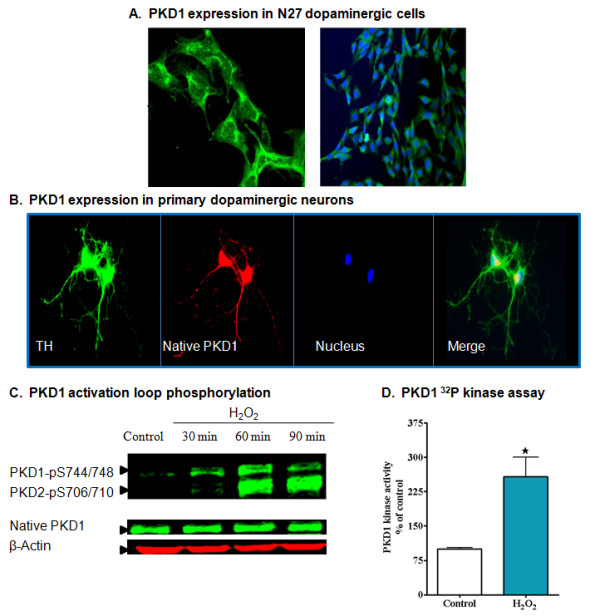
**PKD1 is highly expressed in dopaminergic neurons and activated during initial stages of H_2_O_2 _-induced oxidative stress**. Immunofluorescence analysis of N27 dopaminergic cells stained for native PKD1 using fluorescence and confocal microscopy. Nuclei were stained with Hoechst dye (A). Primary dopaminergic neurons staining for tyrosine hydroxylase (TH) obtained from the mouse substantia nigral region show co-localization of native PKD1 with TH. TH - Green, PKD1 native - Red, Nucleus - Blue, Yellow -Merge. Nuclei were stained with Hoechst dye (B). N27 dopaminergic neuronal cells were treated with or without H_2_O_2 _(100 μM) for 30, 60 or 90 min and probed for PKD1 activation loop phosphorylation pS744/pS748 and native PKD1 expression (C). N27 cells were treated with or without H_2_O_2 _(100 μM) and PKD1 kinase activity was measured by [^32^P] kinase assay using syntide 2 substrate at 60 min (D). *, p < 0.05 denotes significant difference between untreated and H_2_O_2 _-treated groups.

We hypothesized that the proteolytically cleaved PKCδ might activate other downstream cell survival signaling molecules to counteract the early stage of oxidative insult. To test this hypothesis, we first used a bioinformatic approach to search for a key pro-survival molecule that interacts with PKCδ. Scansite Motif Scanner software [45] predicted that out of all the PKCs, only PKCδ can phosphorylate the protein kinase D1 (PKD1) activation loop ser residue at high stringency search (additional file 1). Further literature review indicated that PKD1 is an oxidative stress-responsive kinase that can be activated by phosphorylation at the activation loop (S744/S748) in non-neuronal models [22-25,46]. This encouraged us to look further for the expression and activation of PKD1 in dopaminergic neuronal cells. Immunocytochemical staining showed abundant expression of PKD1 in N27 dopaminergic cells, as visualized by confocal and fluorescence microscopy (Figure 2A), which is similar to the expression pattern of PKCδ in this cell type. Importantly, PKD1 is also highly expressed in primary tyrosine hydroxylase (TH) positive dopaminergic neurons obtained from mouse substantia nigra in the cytosolic region (Figure 2B).

To determine if oxidative stress can induce PKD1 Ser 744/Ser 748 phosphorylation in the activation loop, we examined the ability of H_2_O_2 _to induce time-dependent PKD1 activation loop phosphorylation in N27 dopaminergic cells. As shown in Figure 2C, 100 μM H_2_O_2 _induced transient PKD1 activation loop phosphorylation corresponding to the 120 kDa band starting at around 30 min, peaking at 60 min and returning to control levels after 90 min, with native protein levels remaining the same. We also observed a second band around 100 kDa, which might correspond to the other isoform, PKD2. According to the manufacturers (Cell Signaling Technology), the phospho-specific antibody can also detect PKD2 Ser 706/Ser 710 phosphorylation because of the conserved activation loop residues between PKD isoforms. However, the PKD2 activation loop phosphorylation does not follow the transient pattern of activation observed with PKD1 (Figure 2C). Furthermore, the activation loop phosphorylation of PKD1 increased the PKD1 kinase activity, as measured by a [^32^P] kinase assay using Syntide 2 substrate (Figure 2D). Collectively, these results demonstrate that oxidative stress activates PKD1 at early stages through phosphorylation of the dual phospho sites pS744/pS748.

### Oxidative stress-induced PKD1 activation depends on PKCδ

**Figure 3:**
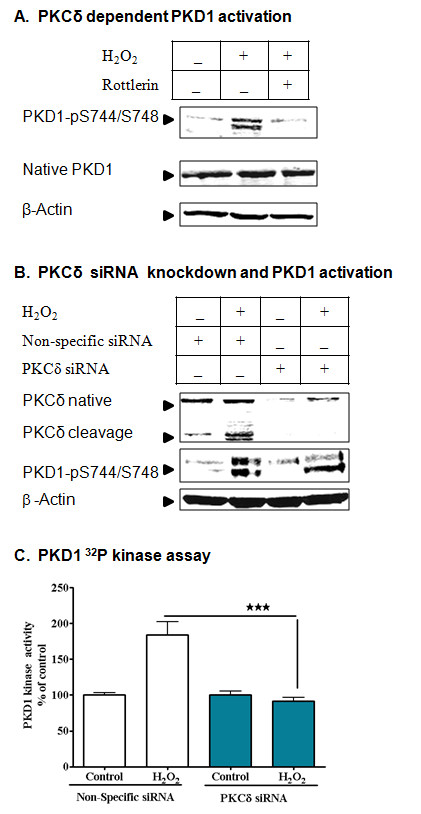
**PKCδ dependent phosphorylation of PKD1 activation loop**. N27 dopaminergic cells were treated with H_2_O_2 _(100 μM) with or without 1 μM rottlerin, and the lysates were probed for PKD1 activation phosphorylation (A). N27 dopaminergic cells were transfected with 1 μM PKCδ siRNA and non-specific siRNA and monitored for PKCδ protein expression and PKD1 activation loop phosphorylation after H_2_O_2 _treatment (B) and PKD1 kinase activity assay was performed. **, p < 0.01 denotes significant difference between NS-siRNA-H_2_O_2 _and PKCδ-siRNA-H_2_O_2 _groups (C).

To further determine whether PKCδ regulates PKD1, we used both pharmacological and genetic approaches to suppress the PKCδ and then measured the level of PKD1 activation. Treatment of N27 cells with the PKCδ inhibitor rottlerin (1 μM) completely suppressed H_2_O_2 _-induced PKD1 activation (Figure 3A), suggesting a potential role of PKCδ in PKD1 activation. Next, we used the PKCδ-specific siRNA to knockdown PKCδ and then probed for PKCδ expression and PKD1 phosphorylation. PKCδ knockdown completely attenuated PKD1 activation loop phosphorylation except for the bottom band, corresponding to 100 kDa PKD2 (Figure 3B). Furthermore, PKD1 activity was measured by [^32^P] kinase assay in PKCδ knockdown samples. The suppression in PKCδ expression completely attenuated PKD1 activation during H_2_O_2 _-mediated oxidative stress, confirming that PKCδ is indeed involved in PKD1 activation (Figure 3C). To show the specificity of PKCδ in PKD1 activation in neuronal models, we used PKCα siRNA and then measured PKD1 activation loop phosphorylation (additional file 2). Knockdown of PKCα did not attenuate PKD1 activation loop phosphorylation, further demonstrating the specificity of PKCδ-mediated PKD1 phosphorylation in dopaminergic neuronal models.

### The constitutively active catalytic fragment of PKCδ mediates PKD1 activation

**Figure 4:**
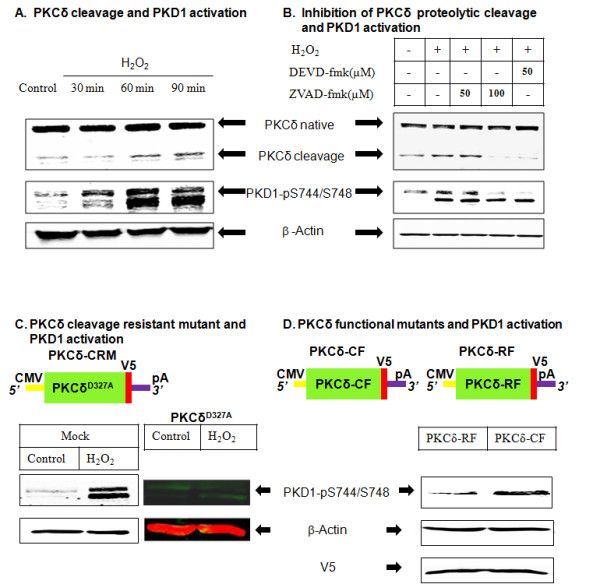
**Proteolytically activated PKCδ-CF contributes to PKD1 phosphorylation**. N27 dopaminergic cells treated with H_2_O_2 _(100 μM) for 30, 60 or 90 minutes were monitored for PKCδ-CF (A). N27 dopaminergic cells were treated with H_2_O_2 _± DEVD-fmk (50 μM) and ± ZVAD-fmk (50 μM and 100 μM) for 60 min and monitored for PKD1 activation and PKCδ cleavage (B). N27 dopaminergic cells stably expressing the cleavage-resistant mutant of PKCδ (PKCδ^D327A^) were treated with H_2_O_2 _and monitored for PKD1 activation (C). PKD1 activation was monitored in N27 dopaminergic cells transfected with the catalytic fragment of PKCδ (PKCδ-CF) and the regulatory fragment of PKCδ (PKCδ-RF). Additionally, the mock transfection group treated with or without H_2_O_2 _was also monitored for PKD1 activation (D).

Time course analysis of PKCδ proteolytic cleavage and PKD1 activation revealed that the onset of proteolytic activation of PKCδ coincides with the maximal activation of PKD1 at 60 min following H_2_O_2_-induced oxidative stress (Figure 4A). Previously, we had shown that caspase-3 inhibitor z-DEVD-fmk and pan caspase inhibitor ZVAD-fmk attenuate H_2_O_2_-induced proteolytic activation of PKCδ. Therefore, we examined whether the proteolytically activated PKCδ contributes to PKD1 activation. As shown in Figure 4B, co-treatment with z-DEVD-fmk and ZVAD-fmk for 1 h significantly blocked H_2_O_2_-induced activation loop phosphorylation of PKD1 as well as proteolytic cleavage of PKCδ. Coincidentally, H_2_O_2_-induced activation loop phosphorylation of PKD2 was not affected in the presence of these caspase inhibitors. To further confirm the role of proteolytically activated PKCδ in PKD1 activation, we used N27 cells stably expressing the PKCδ cleavage-resistant mutant PKCδ^D327A ^(PKCδ-CRM). H_2_O_2 _treatment in PKCδ-CRM cells significantly attenuated PKD1 phosphorylation, confirming that the proteolytically cleaved PKCδ contributes to PKD1 phosphorylation during early stages of oxidative stress (Figure 4C). To further support this observation, we separately over-expressed the PKCδ catalytic fragment (V5-PKCδ-CF) and PKCδ regulatory fragment (V5-PKCδ-RF) using a lentiviral vector (plenti6/V5-D-TOPO) in N27 cells and then evaluated for activation of PKD1. Interestingly, V5-PKCδ-CF over-expressing cells had increased PKD1 phosphorylation, while V5-PKCδ-RF over-expressing cells did not increase phospho-PKD1 levels (Figure 4D). Collectively, these results demonstrate that the constitutively active catalytic fragment of PKCδ mediates PKD1 activation loop phosphorylation.

### PKD1 activation functions as an anti-apoptotic protective mechanism against oxidative stress

**Figure 5:**
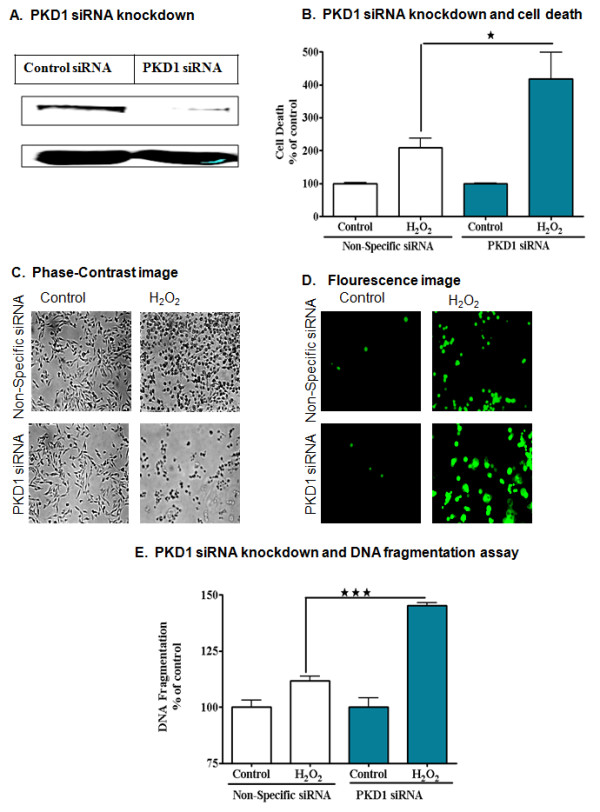
**PKD1 has a cell survival function during oxidative stress in dopaminergic neuronal cells**. N27 dopaminergic cells were transfected with 1 μM PKD1 siRNA and non-specific siRNA (A) and treated with 100 μM H_2_O_2 _for 120 minutes and monitored for cytotoxicity using Sytox green dye. Fluorescence measurements for the incorporation of Sytox green read using a flourescence plate reader (B) and visualised by phase contrast and fluorescence microscopy (C&D). DNA fragmentation assay (E) showed increased cytotoxicity and apoptosis in PKD1 knocked down samples exposed to H_2_O_2_. *, p < 0.05 and ***, p < 0.001 denote significant difference between Non-Specific siRNA- H_2_O_2 _and PKD1 siRNA-H_2_O_2 _treated groups.

We previously reported that proteolytically activated PKCδ (PKCδ-CF) mediates apoptosis in neurotoxicity models [15,19,47] and therefore, we initially hypothesized that PKCδ proteolytic cleavage-dependent activation of PKD1 may have a proapoptotic function. Surprisingly, PKD1 knockdown siRNA significantly augmented H_2_O_2 _-induced cell death at 2 h, as measured by Sytox cell death assay (Figure 5A). PKD1 knockdown increased cell death by nearly twofold compared to control groups during H_2_O_2 _treatment, indicating a pro-survival role for PKD1 against oxidative stress (Figure 5B). Visualization of PKD1 knockdown cells by phase contrast and fluorescence microscopy further confirmed that PKD1 knockdown cells are more sensitive than non-specific control cells to H_2_O_2_-induced cytotoxicity (Figure 5C and 5D). We further measured the H_2_O_2 _-induced apoptosis in PKD1 knockdown cells by DNA fragmentation ELISA assay at 3 h. The results showed an increase in DNA fragmentation in the PKD1 knockdown group, as compared to the non-specific siRNA control group, further confirming the anti-apoptotic role of PKD1 in dopaminergic cells (Figure 5E).

### PKD1 C-terminal Ser 916 phosphorylation precedes PKD1 Ser 744/Ser 748 activation loop phosphorylation during oxidative stress

**Figure 6:**
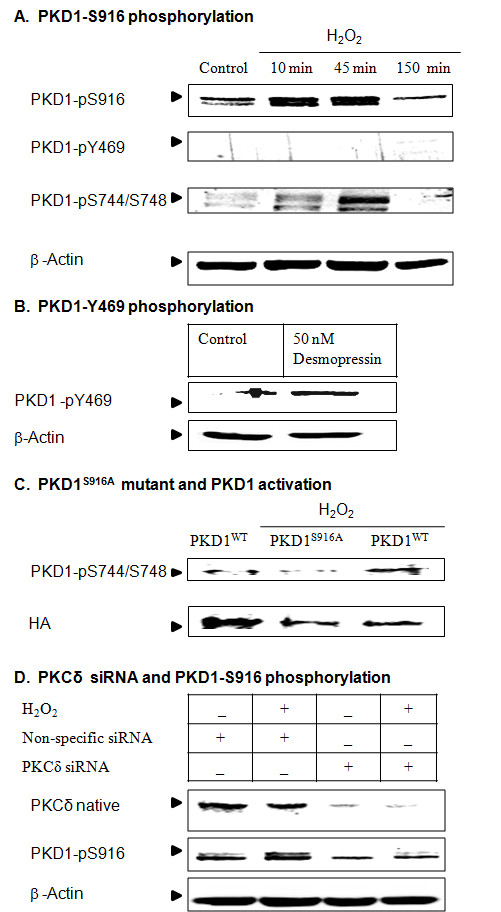
**PKCδ-dependent phosphorylation of PKD1 at S916 site precedes PKD1 S744/S748 active loop phosphorylation**. N27 dopaminergic cells were treated with 100 μM H_2_O_2 _for 10-90 min and monitored for PKD1Y469, PKD1 S916 and PKD1 S744/S748 phosphorylation (A). N27 dopaminergic cells were treated with or without 50 nM desmopressin for 1 h and monitored for PKD1 Y469 phosphorylation. The cells show PKD1 Y469 phosphorylation when exposed to desmopressin, while carbachol does not cause PKD1 Y469 phosphorylation (B). N27 cells expressing PKD1^S916A ^mutant blocked PKD1 activation during oxidative stress, as seen by Western blotting for PKD1 S744/S748 phosphorylation and HA expression (C). N27 dopaminergic cells were transfected with 1 μM PKCδ siRNA and non-specific siRNA and monitored for PKCδ protein expression and PKD1 S916 phosphorylation after treatment with or without H_2_O_2 _(D).

We also characterized the sequential mechanisms of PKD1 activation in dopaminergic neuronal cells. We investigated the other two key phosphorylation sites of PKD1, Tyr 469 and Ser 916. As shown in Figure 6A H_2_O_2_-induces transient phosphorylation of PKD1 Ser 916 in a time-dependent manner. The data suggests that PKD1 C-terminal Ser 916 was rapidly phosphorylated as early as 10 min after H_2_O_2 _treatment continues to increase up to 90 min and decreases at 150 min (Figure 6A). The phospho PKD1-pS916 antibody detects a doublet PKD1 band. On the other hand, H2O2 failed to induce PKD1-tyr 469 phosphorylation in N27 dopaminergic cells (Figure 6A). Our results are consistent with a previous study suggesting that PKD1 tyr 469 phosphorylation does not occur in H_2_O_2_-treated Swiss 3T3 cells [48]. Similarly, it was shown that Vasopressin a GPCR agonist can activates PKD1 in Swiss 3T3 cells [30]. Since H_2_O_2 _did not phosphorylate tyr 469, we used desmopressin, a synthetic analogue of vasopressin to induce tyr 469 phosphorylation. Exposure of N27 dopaminergic cells to the positive control desmopressin induced tyr 469 phosphorylation (Figure 6B). To further evaluate the involvement of PKD1 Ser 916 phosphorylation in PKD1 activation, we overexpressed the PKD1^S916A ^mutant and PKD1^WT ^plasmids in dopaminergic cells and then stimulated the cells with H_2_O_2_. Overexpression of PKD1^S916A ^mutant blocked PKD1 activation loop phosphorylation (Figure 6C), suggesting that Ser 916 phosphorylation is an early event that has to occur prior to PKD1 activation loop phosphorylation. Furthermore, knockdown of PKCδ attenuated PKD1^S916 ^phosphorylation, demonstrating that this is PKCδ dependent (Figure 6D). Together, these results suggest that PKD1 Ser 744/Ser 748 activation loop phosphorylation is intrinsically regulated by PKCδ via C-terminal phosphorylation of PKD1 Ser916.

### PKD1 activation acts as a protective compensatory mechanism during early stages of oxidative stress

**Figure 7:**
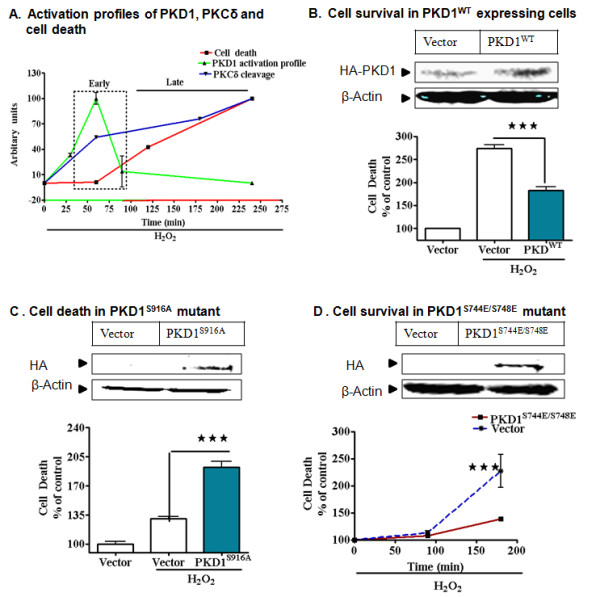
**PKD1 activation acts as an early protective compensatory mechanism**. A comparative time course graph based on quantifying PKD1 activation profile, PKCδ cleavage profile and cytotoxicity during H_2_O_2 _exposure (A). N27 dopaminergic cells transiently transfected with 5 μM full length PKD1 plasmid (PKD1^WT^) and 5 μM vector plasmid were treated with or without 100 μM H_2_O_2 _for 150 minutes and monitored for cytotoxicity using sytox green; PKD1 protected against cytotoxicity (B). N27 dopaminergic cells transiently transfected with 5 μM PKD1^S916A ^plasmid and 5 μM vector plasmid were treated with or without 100 μM H_2_O_2 _for 150 minutes and monitored for cytotoxicity using sytox green; increased cytotoxicity was observed in the cells (C). N27 dopaminergic cells transiently transfected with 5 μM PKD1^S744E/S748E ^and 5 μM vector plasmid (D) were treated with or without 100 μM H_2_O_2 _and monitored for cytotoxicity at various time points using sytox green. ***, p < 0.001 denotes significant difference between treatment groups from n≥6.

To understand the relationship between PKCδ/PKD1 activation and neuronal cell death during oxidative stress, we quantified the PKCδ/PKD1 activation profile from the Western blots and then compared them with the profile of neuronal cell death during H_2_O_2_-induced oxidative stress. As shown in Figure 7A, PKD1 was activated at the early stages of oxidative insult and no measurable cell death was observed until PKD1 activation started declining to basal levels. Alternatively, PKCδ activation was concomitantly increased along with H_2_O_2 _-induced cytotoxic cell death at the later stage of oxidative stress. Thus, the inverse correlation of PKD1 activation with cytotoxicity suggests that PKD1 activation may act as a compensatory protective response during early stages of oxidative insult. To test this hypothesis, we first overexpressed the full-length human PKD1 (PKD1^WT^) in N27 cells and then examined the H_2_O_2 _-induced cytotoxicity. As anticipated, PKD1^WT ^overexpression protected the dopaminergic cells against oxidative stress-induced cytotoxicity at 2 h (Figure 7B). In order to establish the pro-survival function of PKD1 activation, we examined whether overexpression of the PKD1^S916A ^phosphorylation defective mutant exacerbated H_2_O_2 _-induced neuronal cell death. As shown in Figure 7C, overexpression of the PKD1^S916A ^phosphorylation defective mutant exacerbated H_2_O_2 _-induced cytotoxic cell death as early as 90 minutes, compared to vector overexpressing cells. To further confirm our hypothesis that PKD1 activation loop phosphorylation acts as an early protective compensatory response, we overexpressed the activation loop active plasmid (PKD1^S744E^/^S748E^), where the replacement of serine with glutamate makes the kinase constitutively active [49], and cytotoxicity was monitored for up to 3h following H_2_O_2 _treatment (Figure 7D). The constitutively active PKD1 mutants tremendously suppressed the cytotoxicity, even during late stages of oxidative insult, indicating that PKD1 activation is a very significant early protective compensatory mechanism in dopaminergic cells. Collectively, these results demonstrate that PKD1 is a cell survival kinase that is activated during the early stages of oxidative stress to protect against cytotoxicity in dopaminergic cell models.

### Activated PKD1 translocates to nucleus during oxidative stress in cell culture models of neurodegeneration

**Figure 8:**
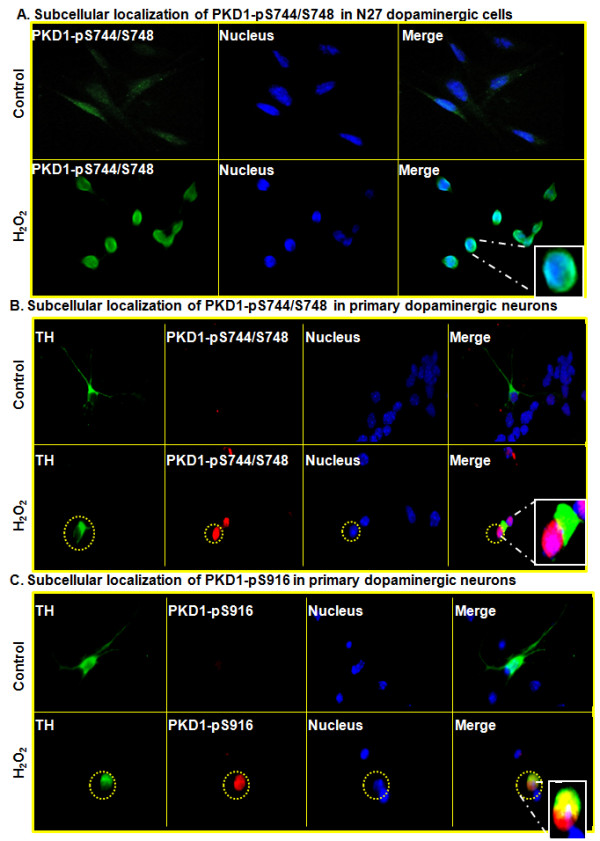
**Activated PKD1 translocates to nucleus during H_2_O_2 _-induced oxidative stress**. Immunofluorescence analysis of N27 dopaminergic cells stained for activated PKD1 using fluorescence microscopy during H_2_O_2 _exposure show translocation to nucleus. PKD1pS744/S748 - Green, Nucleus - Blue. Nuclei were stained with Hoechst dye (A). Immunofluorescence analysis of primary dopaminergic neurons staining for TH obtained from the mouse substantia nigral region shows translocation of activated PKD1 to the nucleus during H_2_O_2 _exposure.TH-Green, PKD1pS744/S748 - Red, Nucleus - Blue, Merge - Pink. Nuclei were stained with Hoechst dye (B). Primary dopaminergic neurons staining for TH show presence of PKD1pS916 in both cytosol and nucleus during H_2_O_2 _exposure. TH - Green, PKD1pS916- Red, Nucleus - Blue, Pink - Merge in nucleus, yellow -Merge in cytosol. Nuclei were stained with Hoechst dye (C).

We also performed immunocytochemical staining to examine the subcellular localization of activated PKD1 during oxidative stress. Activated PKD1 (PKD1pS744/pS748) (green) co-localized with the nuclear Hoechst stain (blue) at 1 h during H_2_O_2 _-induced oxidative stress in N27 cells, as visualized by fluorescence microscopy (Figure 8A). These results indicate that activated PKD1 translocates to the nucleus of dopaminergic neuronal cells to carry out cell repair and survival functions.

Furthermore, we examined oxidative stress-induced PKD1 activation in primary mesencephalic dopaminergic neurons. Mouse primary mesencephalic neuronal cultures were treated with a low dose of 10 μM H_2_O_2 _to induce oxidative stress and then subcellular localization of PKD1 activation was monitored by TH/PKD1 double immunolabeling. Activated PKD1 (red) (PKD1 pS744/pS748) co-localized (pink) with the nuclear Hoechst stain (blue) following H_2_O_2 _treatment in primary mesencephalic neurons staining for TH (green), as visualized by fluorescence microscopy (Figure 8B). We also examined the activation profile of PKD1 pS916 C-terminal phosphorylation. PKD1pS916 (red) was localized (pink/yellow) in both cytosol and nucleus of TH +ve primary mesencephalic neurons staining for green during H_2_O_2 _-induced oxidative stress (Figure 8C).

### Parkinsonian-specific toxicant causes PKD1 activation

**Figure 9:**
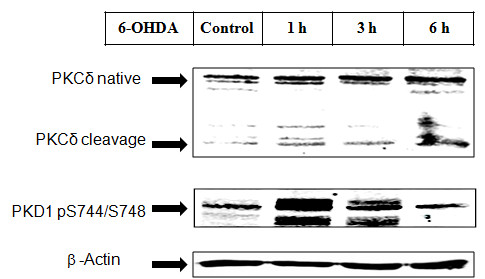
**PKD1 is activated by 6-OHDA induced oxidative stress**. N27 dopaminergic neuronal cells were treated with or without 6-OHDA (100 μM) for 1, 3, and 6 h and probed for PKD1 activation loop phosphorylation pS744/pS748 and PKCδ cleavage.

Activation of this signaling pathway was also tested using the parkinsonian-specific toxicant 6-OHDA. We treated N27 cells with 100 μM 6-OHDA and performed a time-course analysis for 1, 3, and 6 h. PKD1 activation started as early as 1 h and continued until 3 h before reaching control levels at around 6 h (Figure 9). This further confirmed the involvement of PKD1 signaling during parkinsonian-specific oxidative insult.

## Discussion

The present study reveals a novel protective compensatory signaling mechanism via PKCδ-PKD1 molecular interaction in dopaminergic neuronal cells. Through our collective results, we report for the first time four key findings in a dopaminergic neuronal model pertinent to oxidative stress-mediated neurodegenerative processes: (i) A proteolytically activated catalytic PKCδ fragment (PKCδ-CF) phosphorylates and activates protein kinase D1 (PKD1); (ii) PKD1 activation counteracts early stage oxidative damage and protects dopaminergic neuronal cells from cytotoxicity; (iii) PKCδ-dependent phosphorylation of ser 916 residue precedes ser 744/ser 748; (iv) PKCδ - PKD1 crosstalk tightly regulates cell survival and cell death to maintain cellular homeostasis in response to oxidative damage. The elucidation of this compensatory signal transduction mechanism in neurodegenerative diseases may enhance understanding of degenerative processes and lead to development of novel treatment modalities.

H_2_O_2_-induced cytotoxicity causes apoptosis in neuronal and non-neuronal cells [15,43,44,50]. Generally, oxidative stress-induced apoptosis can be classified into early and late stages. DNA fragmentation occurs in the late stage of apoptosis and is preceded by ROS generation, mitochondrial dysfunction and caspase-3 activation and membrane phosphatidyl exposure [10,47]. In neurodegenerative disorders, especially PD, the signaling mechanisms that contribute to increased vulnerability of dopaminergic neurons to oxidative damage are still under investigation. Most current research focuses on cell death mechanisms in dopaminergic neurons. Some of the signaling kinases responsible for cell death mechanisms in PD include JNK, MLK, MAPK, LRRK2, etc. [51-54]. Earlier, the involvement of a novel biochemical mechanism for cell death in dopaminergic neurons through caspase-mediated proteolytic activation of PKCδ was demonstrated [15-19]. The high levels of persistently active PKCδ catalytic fragment mediate apoptosis during oxidative stress in both cell culture and animal models of PD [15-19]. We also have shown in our earlier study that a positive feedback loop exists during the late stages of oxidative stress, where the persistently active PKCδ catalytic fragment translocates to the mitochondria to promote cytochrome C release and apoptosis [16,17,55].

We previously demonstrated proteolytic activation of PKCδ occurs during the early stages of oxidative stress, even before cell death can occur, and coincides with the initiation of mitochondrial ROS generation/caspase-3 activation in dopaminergic neurons [15,17]. Thus, we speculated that proteolytically activated PKCδ might play a regulatory role during the early stages of apoptosis. Previous research suggests the presence of a variety of protective compensatory mechanisms that counteract the early oxidative insult [8-10]. Since we observed in our present study a significant lag time before induction of cell death during the early stages of oxidative stress (Figure 1B), we hypothesize that proteolytically activated PKCδ might sense the extent of oxidative damage and act as a homeostatic regulator in response to oxidative stress, modulating cell survival and cell death mechanisms through interactions with protective signaling molecules.

Protein kinase D1 (PKD1) is emerging as an important signaling molecule associated with oxidative stress in non-neuronal cell lines [31,35,36]. Studies have shown that oxidative stress increases PKD1 activation loop phosphorylation (pS744/pS748) via full length PKCs, including PKCδ, in non-neuronal models [37,56-59]. However, the functions of PKD1 during oxidative stress-induced neurodegeneration have not been studied previously. In the present study, we report that cleaved active PKCδ phosphorylates the activation loop of PKD1 and activates the kinase during the early stages of H_2_O_2 _-induced oxidative stress in dopaminergic neuronal cells. We also observed a similar activation pattern for PKD1 and PKCδ during oxidative stress caused by the parkinsonian-specific toxicant 6-OHDA (Figure 9). To our knowledge, this is the first report of a novel cell survival/cell death signal regulation by the cleaved catalytic fragment of PKCδ at two different stages of apoptosis based on the extent of oxidative damage.

PKD1 is mainly activated by a diacylglycerol-dependent PKCs mechanism [22,60] or by PKD1 cleavage [61-63]. A recent study shows that PKD1 auto-inhibition is released through phosphorylation at the Y463 site in the regulatory domain, leading to the activation loop phosphorylation by PKCδ full length (PKCδ-FL) in Hela cells [24]. PKD1 is in a closed conformation during the resting stage, with the regulatory fragment having an autoinhibitory effect on the catalytic fragment [46,64]. Multiple phosphorylation sites on PKD1 seem to be important for its activation loop phosphorylation, depending on the cell types and stimuli. In human cancer cell lines, PKD1 can be phosphorylated at multiple sites including Y463, S910 (corresponding to murine Y469, S916) [24,65]. Phosphorylation of Ser 916 (murine) autophosphorylation site correlated with PKD1 activation loop phosphorylation [58,66]. During oxidative stress in non-neuronal models, Tyr 469 is phosphorylated by upstream kinases, which results in release of the Pleckstrin homology (PH) domain autoinhibition prior to activation loop phosphorylation; this mechanism does not involve C-terminus Ser 916 phosphorylation [24,37]. In our dopaminergic neuronal models, oxidative stress failed to induce PKD1 Tyr 469 phosphorylation (Figure 6A), whereas PKD1 Tyr 469 phosphorylation was induced by the positive control desmopressin (Figure 6B). Our results demonstrate that the mechanism of PKD1 activation in dopaminergic neurons is distinct from the mechanisms in other non-neuronal models. We demonstrate that S916 phosphorylation, but not Tyr 469 phosphorylation, is a preceding event that occurs and is required for PKD1ser744/Ser748 activation loop phosphorylation (Figure 6). Our data suggest that Ser 916 phosphorylation on the C-terminal of PKD1 may open the conformation for full activation of the kinase through activation loop phosphorylation during oxidative stress in dopaminergic neurons. A detailed comparative analysis of PKCδ proteolytic activation, PKD1 activation loop phosphorylation and the extent of cell death during oxidative stress revealed an interesting functional relationship between activation of kinases and regulation of cell death. Comparison of PKD1 activation and cytotoxicity shows that PKD1 activation is maximal during the early oxidative stress stage when no measurable cytotoxicity is noted (Figure 7A). Interestingly, when PKD1 activation begins to decline at the end of the early stage, cell death begins to occur. Also, the level of PKCδ proteolytic activation directly correlates with the extent of cell death at the later stage of oxidative stress. When the constitutively active PKD1 mutant (PKD1^S744E^/^S748E^) is overexpressed, dopaminergic cells are resistant to H_2_O_2 _-induced neurotoxicity, even during the late stages of oxidative stress (Figure 7D), which is consistent with our hypothesis that PKD1 activation protects against oxidative damage. The downstream signaling mechanisms of PKD1 activation in dopaminergic neuronal cells are not known. PKD1 translocates to the nucleus and regulates phosphorylation of HDACs and various transcription factors in various non-neuronal cell lines including B cell, cardiomyocytes & oestoblasts [31,23,40,67]. PKD1 translocation to the nucleus after activation in dopaminergic neurons is also noted in the present study (Figure 8), suggesting that nuclear translocation of PKD1 may activate key cell survival transcription factors and genes. Thus, we suggest that PKD1 functions as a cell survival switch and turns 'ON' a protective compensatory mechanism in dopaminergic neurons. Studies are underway to characterize the downstream protective response of PKD1 signaling in nigral dopaminergic neurons.

## Conclusions

**Figure 10:**
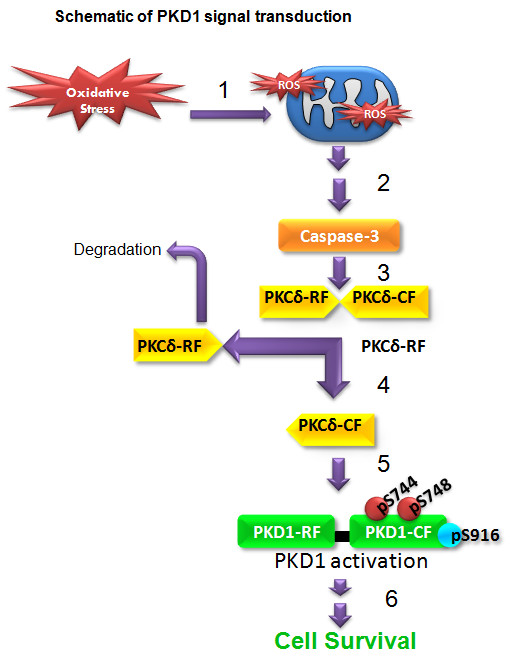
**Schematic of PKCδ-PKD1 signal transduction mechanism during oxidative stress in dopaminergic neuronal cells**. 1) Oxidative stress causes mitochondrial impairment; 2) activation of caspase cascade; 3) caspase-3 mediates proteolytic cleavage of PKCδ; 4) proteolytically cleaved PKCδ-catalytic fragment (CF) is active; 5) PKCδ-CF activates PKD1 by activation loop phosphorylation during the early stage of oxidative stress; 6) Fully active PKD1 regulates cell survival function in N27 dopaminergic cells.

Our results demonstrate that the PKD1-mediated protective mechanism is a novel signal transduction pathway that regulates cell survival and cell death during various stages of oxidative stress in dopaminergic neuronal cells. As depicted in Figure 10, in the early stages of oxidative insult, PKCδ acts as an oxidative stress sensor/regulator and activates PKD1, which serves as a key compensatory protective mechanism against oxidative damage. However, prolonged oxidative insult creates a homeostatic imbalance, causing deactivation of PKD1 and persistent proteolytic activation of PKCδ that contribute to extensive neuronal damage. We previously showed a parallel proapoptotic mechanism involving translocation of PKCδ catalytic fragments to the mitochondria, resulting in a persistent increase in caspase-3 dependent PKCδ proteolytic cleavage via a positive feedback loop mechanism [16,17,55]. Our results suggest that positive modulation of the PKD1-mediated protective mechanism against oxidative damage in dopaminergic neurons may provide novel neuroprotective strategies for treatment of PD.

## Materials and methods

### Cell Culture

The immortalized rat mesencephalic dopaminergic neuronal cell line (N27) was a kind gift from Dr. Kedar N. Prasad (University of Colorado Health Sciences Center, Denver, CO). N27 cells were grown in RPMI 1640 medium containing 10% fetal bovine serum, 2 mm l-glutamine, 50 units of penicillin, and 50 μg/ml streptomycin. Cells were maintained in a humidified atmosphere of 5% CO2 at 37°C, as described previously [16]. N27 cells are used widely as a cell culture model for PD [20,15,21,16,68].

### Primary mesencephalic neuronal culture

Primary mesencephalic neuronal cultures were prepared from the ventral mesencephalon of gestational 16- to 18-day-old mouse embryos, as described earlier [69]. Tissues were dissected from E16 to E18 mouse embryos maintained in ice cold Ca2+-free Hanks' balanced salt solution and then dissociated in Hanks' balanced salt solution containing trypsin-0.25% EDTA for 30 min at 37°C. The dissociated cells were then plated at equal density (0.5 × 106 cells) on 12 mm coverslips precoated with 0.1 mg/ml poly-D-lysine. Cultures were maintained in neurobasal medium fortified with B-27 supplements, 500 μM l-glutamine, 100 IU/ml penicillin, and 100 μg/ml streptomycin (Invitrogen). The cells were maintained in a humidified CO2 incubator (5% CO2 and 37°C). Half of the culture medium was replaced every 2 days. Approximately 6- to 7-day-old cultures were used for experiments. Primary mesencephalic dopaminergic neuronal cells were exposed to 10 μM for 1 h.

### Treatment Paradigm

N27 cells were exposed to H_2_O_2 _(100 μm) for 0-4 h at 37°C. Primary neurons were exposed to H_2_O_2 _(10 μm) for 1 h. Cell lysates were used for Western blotting and immunoprecipitation studies. Untreated cells were grown in the complete medium and used as the experimental control.

### Cytotoxicity Assays

Cell death was determined using the Sytox green cytotoxicity assay, after exposing the N27 cells to H_2_O_2 _(100 μm), as described previously. This cytotoxicity assay was optimized for a multiwell format, which is more efficient and sensitive than other cytotoxicity measurements [70,71]. Briefly, N27 cells were grown in 24-well cell culture plates at 100,000 cells per well and treated with H_2_O_2 _(100 μm) and 1 μm Sytox green fluorescent dye. The Sytox green assay allows dead cells to be viewed directly under a fluorescence microscope, as well as quantitatively measured with a fluorescence microplate reader (excitation 485 nm; emission 538 nm) (Biotek). Phase contrast and fluorescent images were taken after H_2_O_2 _exposure with a NIKON TE2000 microscope, and pictures were captured with a SPOT digital camera.

### ROS Generation Assay

ROS generation was monitored by CM-DCFDA dye, as described previously [15,72]. This is a non-fluorescent dye in its reduced form, but after oxidation in the cells, the acetate group is removed by cellular esterases, resulting in fluorescence. N27 cells were seeded in 48-well plates at a confluence of 40,000 cells/well for 24 h. First, cells were loaded with 10 μM CM-DCFDA dye (Invitrogen) at 37°C for 1 h in the dark. Cells were then treated with H_2_O_2 _in Hanks' balanced salt solution (HBSS) and the fluorescence of the cells was measured using the synergy 2 fluorescence plate reader (Biotek) at various time points (excitation 485 nm; emission 538 nm).

### Immunocytochemistry

The primary mesencephalic neurons or N27 cells after H_2_O_2 _treatment were fixed with 4% paraformaldehyde and processed for immunocytochemical staining. First, nonspecific sites were blocked with 2% bovine serum albumin, 0.5% Triton and 0.05% Tween-20 in phosphate-buffered saline (PBS) for 20 min. The cells then were incubated with antibodies directed against TH, native PKD1 and PKD1-pS744/S748 in PBS containing 1% BSA at 4°C overnight, followed by incubation with Alexa 488 and Alexa 568 conjugated secondary antibodies in PBS containing 1% BSA. Secondary antibody treatments were followed by incubation with Hoechst 33342 dye for 5 min at room temperature to stain the nucleus. The coverslips containing stained cells were washed with PBS, mounted on slides, and viewed under a Nikon inverted fluorescence microscope (model TE-2000U; Nikon, Tokyo, Japan). Both fluorescence and confocal images were captured with a SPOT digital camera (Diagnostic Instruments, Inc., Sterling Heights, MI).

### Western Blot Analysis

Cells were lysed in either modified RIPA buffer or M-PER buffer (Thermo Scientific) for Western blot, immunoprecipitation and kinase assays. Lysates containing equal amounts of protein were loaded in each lane and separated on 10-12% SDS-PAGE, as described previously (Kaul et al., 2003). PKD1 polyclonal (1:1000), PKCδ polyclonal (1: 1000), PKD1-pS744/S748 (1:1000), PKD1-pS916 (1:1000), PKD1-pY469 (1:1000), - and β-actin (1:10000) antibodies were used to blot the membranes. IR dye-800 conjugated anti-rabbit (1:5000) and Alexa Fluor 680 conjugated anti-mouse (1:10000) were used for antibody detection with the Odyssey IR Imaging system (LICOR), as previously described.

### PKCδ Kinase Assay

Immunoprecipitation and PKCδ kinase assay were performed as described earlier [15]. After cell lysis, cell were immunoprecipitated using a polyclonal PKCδ rabbit antibody and protein A Sepharose, and washed three times with PKCδ kinase buffer (40 mM Tris (pH 7.4), 20 mM MgCl_2_, 20 μM ATP, 2.5 mM CaCl2). The reaction was started by adding 20 μl of buffer containing 0.4 mg histone and 5 μCi of [γ-^32^P]ATP (4,500 Ci/mM). After incubation for 10 min at 30°C, SDS loading buffer (2X) was added to the samples to terminate the reaction. The reaction products were separated on SDS-PAGE (12%), and the H1-phosphorylated bands were detected using a phosphoimager (Fujifilm FLA-5100) and quantified with MultiGauge V3.0 software.

### Protein Kinase D1 Kinase Assay

The cells were exposed to H_2_O_2 _(100 μM) for 1 h and cell lysates were immunoprecipitated, as previously reported, with native PKD1 antibody (Santa Cruz). The kinase reaction was carried out at room temperature for 20 min after adding 10 μl of kinase substrate mix (0.1 mM ATP + 10 μci [γ-^32^P] ATP + 2 ug Syntide 2 peptide substrate in kinase buffer). Kinase buffer contains 20 mM Tris pH 7.5, 10 mM MgCl_2_, and 1 mM DTT. The samples were centrifuged to terminate the kinase reaction, and the supernatants containing the phosphorylated peptide were applied as spots to P81 phosphocellulose squares (Whatmann). The papers were washed four times with 0.75% phosphoric acid and once with acetone and dried, and activity was determined by liquid scintillation counting. The samples were also loaded on a SDS-PAGE and probed for native PKD1 to determine equal loading.

### DNA Fragmentation Assay

DNA fragmentation was measured using the Cell Death Detection ELISA Plus assay kit (Roche), for the detection of early apoptotic death, as described previously [15,72]. After 100 μm H_2_O_2 _treatment, the cells were spun down at 200 × g for 5 min and washed once with PBS. Then cells were lysed with lysis buffer provided with the kit. After lysis, the samples were spun down at 10,000 rpm for 10 min to collect the supernatant that was used to measure DNA fragmentation. The supernatants were further dispensed into the microtiter plates coated with streptavidin containing HRP-conjugated antibody cocktail that can detect the nucleosomes. After 2 h incubation, the HRP substrate provided in the kit was added. Measurements were taken in a Synergy 2 multiwell plate reader at 405 nm, with 490 nm as a reference reading.

### Transient and Stable Transfections

cDNA encoding PKCδ catalytic fragment (PKCδ-CF), PKCδ regulatory fragment (PKCδ-RF) and PKCδ caspase-resistant mutant (PKCδ-CRM) (PKCδ^D327A) ^from the pEGFPN1 vector were subcloned into the lentiviral expression vector plenti6/V5-d-TOPO in our lab (herein referred to as V5-PKCδ-CF, V5-PKCδ-RF, V5-PKCδ-CRM) [15,73]. ViraPower Lentiviral Expression System (Invitrogen) was used to establish stable transfections of a caspase-resistant mutant of PKCδ^D327A ^[73]. Full-length human PKD1 plasmid (PKD1-FL), PKD1 activation loop, active PKD1^S744E/S748E ^(PKD1-CA) and PKD1^S916A ^mutants were obtained from Addgene, Inc. [74,49,37]. Electroporation was carried out with an Amaxa Nucleofector transfector instrument, as per the manufacturer's protocol. The transfected cells were then transferred to T-75 flasks or 6-well plates as desired and allowed to grow for a 24 h period before the treatment.

### RNAi

PKCδ-siRNA was prepared by an in vitro transcription method, as described previously [20]. PKCδ-siRNA effectively suppressed > 80% of PKCδ protein expression levels within 24 h post-transfection. Predesigned PKD1-siRNA was purchased from IDT, Inc. PKD1-siRNA effectively suppressed > 80% of PKD1 protein expression levels after 36 h post-transfection. N27 cells (50-70% confluence) were transfected with siRNA duplexes using an Amaxa Nucleofector kit (Amaxa), as described in our previous study [20].

### Statistical Analysis

Data analysis was performed using Prism 3.0 software (GraphPad Software, San Diego, CA). Bonferroni's multiple comparison testing was used to find the significant differences between treatment and control groups. Differences with p < 0.05, p < 0.01, and p < 0.001 were considered significantly different from n ≥ 6 from two or more independent experiments, and are indicated in the figures.

## Abbreviations

PD: Parkinson's disease; PKD1: Protein kinase D1; MAPK: Mitogen-activated protein kinases; PKCδ: Protein kinase C delta; CAMK: Ca ^2+ ^/Calmodulin-Dependent Protein Kinase II; JNK: c-Jun N-terminal kinases; LRRK2: Leucine-rich repeat kinase 2 (LRRK2); MLK: Mixed-lineage kinase; ROS: Reactive oxygen species; MnSOD: Manganese superoxide dismutase; WB: Western Blot; PKC: Protein kinase C; PKCα: Protein kinase C alpha.

## Competing interests

The authors declare that they have no competing interests.

## Authors' contributions

All authors read and approved the final manuscript.

## Supplementary Material

Additional file 1 **Rat PKD1 amino acid sequence obtained from Swiss-Prot database (ID: Q9WTQ1) was analyzed using Scansite Motif software to identify the upstream PKCs that phosphorylate the PKD1 activation loop serine residues**. The analysis done at high stringency shows that only PKCδ phosphorylates PKD1 at the activation loop residue Serine 744 site. Click here for file

Additional file 2 **N27 cells were transfected with 1 μM PKCα siRNA and non-specific siRNA and monitored for PKCα protein expression and PKD1pS744/S748 after H_2_O_2 _treatment**. PKCα knockdown did not cause attenuation in PKD1 activation loop phosphorylation. Click here for file

## References

[B1] Dauer W Przedborski S Parkinson's disease: mechanisms and models Neuron 2003 39 6 889 909 10.1016/S0896-6273(03)00568-3 12971891

[B2] Dawson TM Dawson VL Molecular pathways of neurodegeneration in Parkinson's disease Science 2003 302 5646 819 822 10.1126/science.1087753 14593166

[B3] Di Monte DA The environment and Parkinson's disease: is the nigrostriatal system preferentially targeted by neurotoxins? Lancet Neurol 2003 2 9 531 538 10.1016/S1474-4422(03)00501-5 12941575

[B4] Kanthasamy A Kitazawa M Kaul S Anantharam V Kanthasamy AG A novel oxidative stress-dependent apoptotic pathway in pesticide-induced dopaminergic degeneration: relevance to environmental factors and Parkinson's disease J Neurochem 2002 81 suppl 1 76

[B5] Przedborski S Pathogenesis of nigral cell death in Parkinson's disease Parkinsonism Relat Disord 2005 11 Suppl 1 S3 7 15885625 10.1016/j.parkreldis.2004.10.012

[B6] Veurink G Fuller SJ Atwood CS Martins RN Genetics, lifestyle and the roles of amyloid beta and oxidative stress in Alzheimer's disease Ann Hum Biol 2003 30 6 639 667 10.1080/03014460310001620144 14675907

[B7] Malkus KA Tsika E Ischiropoulos H Oxidative modifications, mitochondrial dysfunction, and impaired protein degradation in Parkinson's disease: how neurons are lost in the Bermuda triangle Mol Neurodegener 2009 4 24 10.1186/1750-1326-4-24 19500376 PMC2701947

[B8] Moreira PI Zhu X Liu Q Honda K Siedlak SL Harris PL Smith MA Perry G Compensatory responses induced by oxidative stress in Alzheimer disease Biol Res 2006 39 1 7 13 16629160 10.4067/s0716-97602006000100002

[B9] Moreira PI Zhu X Lee HG Honda K Smith MA Perry G The (un)balance between metabolic and oxidative abnormalities and cellular compensatory responses in Alzheimer disease Mech Ageing Dev 2006 127 6 501 506 10.1016/j.mad.2006.01.024 16516950

[B10] Chong ZZ Li F Maiese K Oxidative stress in the brain: novel cellular targets that govern survival during neurodegenerative disease Prog Neurobiol 2005 75 3 207 246 10.1016/j.pneurobio.2005.02.004 15882775

[B11] Andersen JK Oxidative stress in neurodegeneration: cause or consequence? Nat Med 2004 10 Suppl S18 25 15298006 10.1038/nrn1434

[B12] Jenner P The contribution of the MPTP-treated primate model to the development of new treatment strategies for Parkinson's disease Parkinsonism Relat Disord 2003 9 3 131 137 10.1016/S1353-8020(02)00115-3 12573867

[B13] Tan S Wood M Maher P Oxidative stress induces a form of programmed cell death with characteristics of both apoptosis and necrosis in neuronal cells J Neurochem 1998 71 1 95 105 9648855 10.1046/j.1471-4159.1998.71010095.x

[B14] Przedborski S Vila M The 1-methyl-4-phenyl-1,2,3,6-tetrahydropyridine mouse model: a tool to explore the pathogenesis of Parkinson's disease Ann N Y Acad Sci 2003 991 189 198 12846987

[B15] Kaul S Anantharam V Yang Y Choi CJ Kanthasamy A Kanthasamy AG Tyrosine phosphorylation regulates the proteolytic activation of protein kinase Cdelta in dopaminergic neuronal cells J Biol Chem 2005 280 31 28721 28730 10.1074/jbc.M501092200 15961393

[B16] Anantharam V Kitazawa M Wagner J Kaul S Kanthasamy AG Caspase-3-dependent proteolytic cleavage of protein kinase Cdelta is essential for oxidative stress-mediated dopaminergic cell death after exposure to methylcyclopentadienyl manganese tricarbonyl J Neurosci 2002 22 5 1738 1751 11880503 10.1523/JNEUROSCI.22-05-01738.2002 PMC6758879

[B17] Kitazawa M Anantharam V Kanthasamy AG Dieldrin induces apoptosis by promoting caspase-3-dependent proteolytic cleavage of protein kinase Cdelta in dopaminergic cells: relevance to oxidative stress and dopaminergic degeneration Neuroscience 2003 119 4 945 964 10.1016/S0306-4522(03)00226-4 12831855

[B18] Sun F Kanthasamy A Anantharam V Kanthasamy AG Environmental neurotoxic chemicals-induced ubiquitin proteasome system dysfunction in the pathogenesis and progression of Parkinson's disease Pharmacol Ther 2007 114 3 327 344 10.1016/j.pharmthera.2007.04.001 17521740

[B19] Zhang D Anantharam V Kanthasamy A Kanthasamy AG Neuroprotective effect of protein kinase C delta inhibitor rottlerin in cell culture and animal models of Parkinson's disease J Pharmacol Exp Ther 2007 322 3 913 922 10.1124/jpet.107.124669 17565007

[B20] Yang Y Kaul S Zhang D Anantharam V Kanthasamy AG Suppression of Caspase-3-dependent proteolytic activation of protein kinase C-delta by small interfering RNA prevents MPP+-induced dopaminergic degeneration Molecular and Cellular Neuroscience 2004 25 3 406 421 10.1016/j.mcn.2003.11.011 15033169

[B21] Kanthasamy AG Anantharam V Zhang D Latchoumycandane C Jin H Kaul S Kanthasamy A A novel peptide inhibitor targeted to caspase-3 cleavage site of a proapoptotic kinase protein kinase C delta (PKCδelta) protects against dopaminergic neuronal degeneration in Parkinson's disease models Free Radic Biol Med 2006 41 10 1578 1589 10.1016/j.freeradbiomed.2006.08.016 17045926

[B22] Zugaza JL Sinnett-Smith J Van Lint J Rozengurt E Protein kinase D (PKD) activation in intact cells through a protein kinase C-dependent signal transduction pathway Embo J 1996 15 22 6220 6230 8947045 PMC452445

[B23] Rey O Sinnett-Smith J Zhukova E Rozengurt E Regulated nucleocytoplasmic transport of protein kinase D in response to G protein-coupled receptor activation J Biol Chem 2001 276 52 49228 49235 10.1074/jbc.M109395200 11641411

[B24] Storz P Doppler H Johannes FJ Toker A Tyrosine phosphorylation of protein kinase D in the pleckstrin homology domain leads to activation J Biol Chem 2003 278 20 17969 17976 10.1074/jbc.M213224200 12637538

[B25] Van Lint J Ni Y Valius M Merlevede W Vandenheede JR Platelet-derived growth factor stimulates protein kinase D through the activation of phospholipase Cgamma and protein kinase C J Biol Chem 1998 273 12 7038 7043 10.1074/jbc.273.12.7038 9507012

[B26] Matthews SA Liu P Spitaler M Olson EN McKinsey TA Cantrell DA Scharenberg AM Essential role for protein kinase D family kinases in the regulation of class II histone deacetylases in B lymphocytes Mol Cell Biol 2006 26 4 1569 1577 10.1128/MCB.26.4.1569-1577.2006 16449666 PMC1367196

[B27] Jamora C Yamanouye N Van Lint J Laudenslager J Vandenheede JR Faulkner DJ Malhotra V Gbetagamma-mediated regulation of Golgi organization is through the direct activation of protein kinase D Cell 1999 98 1 59 68 10.1016/S0092-8674(00)80606-6 10412981

[B28] Haussermann S Kittstein W Rincke G Johannes FJ Marks F Gschwendt M Proteolytic cleavage of protein kinase Cmu upon induction of apoptosis in U937 cells FEBS Lett 1999 462 3 442 446 10.1016/S0014-5793(99)01577-X 10622742

[B29] Prigozhina NL Waterman-Storer CM Protein kinase D-mediated anterograde membrane trafficking is required for fibroblast motility Curr Biol 2004 14 2 88 98 10.1016/j.cub.2004.01.003 14738729

[B30] Zhukova E Sinnett-Smith J Rozengurt E Protein kinase D potentiates DNA synthesis and cell proliferation induced by bombesin, vasopressin, or phorbol esters in Swiss 3T3 cells J Biol Chem 2001 276 43 40298 40305 11514571 10.1074/jbc.M106512200

[B31] Storz P Doppler H Toker A Protein kinase D mediates mitochondrion-to-nucleus signaling and detoxification from mitochondrial reactive oxygen species Mol Cell Biol 2005 25 19 8520 8530 10.1128/MCB.25.19.8520-8530.2005 16166634 PMC1265746

[B32] Sidorenko SP Law CL Klaus SJ Chandran KA Takata M Kurosaki T Clark EA Protein kinase C mu (PKC mu) associates with the B cell antigen receptor complex and regulates lymphocyte signaling Immunity 1996 5 4 353 363 10.1016/S1074-7613(00)80261-7 8885868

[B33] Hurd C Waldron RT Rozengurt E Protein kinase D complexes with C-Jun N-terminal kinase via activation loop phosphorylation and phosphorylates the C-Jun N-terminus Oncogene 2002 21 14 2154 2160 10.1038/sj.onc.1205290 11948398

[B34] Wang Y Waldron RT Dhaka A Patel A Riley MM Rozengurt E Colicelli J The RAS effector RIN1 directly competes with RAF and is regulated by 14-3-3 proteins Mol Cell Biol 2002 22 3 916 926 10.1128/MCB.22.3.916-926.2001 11784866 PMC133556

[B35] Song J Li J Qiao J Jain S Mark Evers B Chung DH PKD prevents H2O2-induced apoptosis via NF-kappaB and p38 MAPK in RIE-1 cells Biochem Biophys Res Commun 2009 378 3 610 614 10.1016/j.bbrc.2008.11.106 19059215 PMC2631172

[B36] Storz P Mitochondrial ROS--radical detoxification, mediated by protein kinase D Trends Cell Biol 2007 17 1 13 18 10.1016/j.tcb.2006.11.003 17126550

[B37] Storz P Doppler H Toker A Activation loop phosphorylation controls protein kinase D-dependent activation of nuclear factor kappaB Mol Pharmacol 2004 66 4 870 879 10.1124/mol.104.000687 15226414

[B38] Fielitz J Kim MS Shelton JM Qi X Hill JA Richardson JA Bassel-Duby R Olson EN Requirement of protein kinase D1 for pathological cardiac remodeling Proc Natl Acad Sci USA 2008 105 8 3059 3063 10.1073/pnas.0712265105 18287012 PMC2268584

[B39] Evans IM Britton G Zachary IC Vascular endothelial growth factor induces heat shock protein (HSP) 27 serine 82 phosphorylation and endothelial tubulogenesis via protein kinase D and independent of p38 kinase Cell Signal 2008 20 7 1375 1384 10.1016/j.cellsig.2008.03.002 18440775

[B40] Parra M Kasler H McKinsey TA Olson EN Verdin E Protein kinase D1 phosphorylates HDAC7 and induces its nuclear export after T-cell receptor activation J Biol Chem 2005 280 14 13762 13770 10.1074/jbc.M413396200 15623513

[B41] Besirli CG Johnson EM Jr The activation loop phosphorylation of protein kinase D is an early marker of neuronal DNA damage J Neurochem 2006 99 1 218 225 10.1111/j.1471-4159.2006.04116.x 16911582

[B42] Bisbal M Conde C Donoso M Bollati F Sesma J Quiroga S Diaz Anel A Malhotra V Marzolo MP Caceres A Protein kinase d regulates trafficking of dendritic membrane proteins in developing neurons J Neurosci 2008 28 37 9297 9308 10.1523/JNEUROSCI.1879-08.2008 18784310 PMC2648138

[B43] Chang H Oehrl W Elsner P Thiele JJ The role of H2O2 as a mediator of UVB-induced apoptosis in keratinocytes Free Radic Res 2003 37 6 655 663 10.1080/1071576031000094907 12868492

[B44] Kumar S Bharti A Mishra NC Raina D Kharbanda S Saxena S Kufe D Targeting of the c-Abl tyrosine kinase to mitochondria in the necrotic cell death response to oxidative stress J Biol Chem 2001 276 20 17281 17285 10.1074/jbc.M101414200 11350980

[B45] Obenauer JC Cantley LC Yaffe MB Scansite 2.0: Proteome-wide prediction of cell signaling interactions using short sequence motifs Nucleic Acids Res 2003 31 13 3635 3641 10.1093/nar/gkg584 12824383 PMC168990

[B46] Iglesias T Rozengurt E Protein kinase D activation by mutations within its pleckstrin homology domain J Biol Chem 1998 273 1 410 416 10.1074/jbc.273.1.410 9417097

[B47] Kaul S Kanthasamy A Kitazawa M Anantharam V Kanthasamy AG Caspase-3 dependent proteolytic activation of protein kinase C delta mediates and regulates 1-methyl-4-phenylpyridinium (MPP+)-induced apoptotic cell death in dopaminergic cells: relevance to oxidative stress in dopaminergic degeneration Eur J Neurosci 2003 18 6 1387 1401 10.1046/j.1460-9568.2003.02864.x 14511319

[B48] Waldron RT Rey O Zhukova E Rozengurt E Oxidative stress induces protein kinase C-mediated activation loop phosphorylation and nuclear redistribution of protein kinase D J Biol Chem 2004 279 26 27482 27493 10.1074/jbc.M402875200 15084589

[B49] Storz P Doppler H Toker A Protein kinase Cdelta selectively regulates protein kinase D-dependent activation of NF-kappaB in oxidative stress signaling Mol Cell Biol 2004 24 7 2614 2626 10.1128/MCB.24.7.2614-2626.2004 15024053 PMC371115

[B50] Carvour M Song C Kaul S Anantharam V Kanthasamy A Chronic low-dose oxidative stress induces caspase-3-dependent PKCδelta proteolytic activation and apoptosis in a cell culture model of dopaminergic neurodegeneration Ann N Y Acad Sci 2008 1139 197 205 10.1196/annals.1432.020 18991865 PMC2657189

[B51] Greggio E Bisaglia M Civiero L Bubacco L Leucine-rich repeat kinase 2 and alpha-synuclein: intersecting pathways in the pathogenesis of Parkinson's disease? Mol Neurodegener 6 1 6 10.1186/1750-1326-6-6 PMC3035023 21244648

[B52] Choi WS Eom DS Han BS Kim WK Han BH Choi EJ Oh TH Markelonis GJ Cho JW Oh YJ Phosphorylation of p38 MAPK induced by oxidative stress is linked to activation of both caspase-8- and -9-mediated apoptotic pathways in dopaminergic neurons J Biol Chem 2004 279 19 20451 20460 10.1074/jbc.M311164200 14993216

[B53] Luo Y Umegaki H Wang X Abe R Roth GS Dopamine induces apoptosis through an oxidation-involved SAPK/JNK activation pathway J Biol Chem 1998 273 6 3756 3764 10.1074/jbc.273.6.3756 9452508

[B54] Wang D Tang B Zhao G Pan Q Xia K Bodmer R Zhang Z Dispensable role of Drosophila ortholog of LRRK2 kinase activity in survival of dopaminergic neurons Mol Neurodegener 2008 3 3 10.1186/1750-1326-3-3 18257932 PMC2276501

[B55] Kanthasamy AG Kitazawa M Kanthasamy A Anantharam V Role of proteolytic activation of protein kinase Cdelta in oxidative stress-induced apoptosis Antioxid Redox Signal 2003 5 5 609 620 10.1089/152308603770310275 14580317

[B56] Yuan J Bae D Cantrell D Nel AE Rozengurt E Protein kinase D is a downstream target of protein kinase Ctheta Biochem Biophys Res Commun 2002 291 3 444 452 10.1006/bbrc.2002.6469 11855809

[B57] Waldron RT Rozengurt E Protein kinase C phosphorylates protein kinase D activation loop Ser744 and Ser748 and releases autoinhibition by the pleckstrin homology domain J Biol Chem 2003 278 1 154 163 12407104 10.1074/jbc.M208075200

[B58] Brandlin I Hubner S Eiseler T Martinez-Moya M Horschinek A Hausser A Link G Rupp S Storz P Pfizenmaier K Protein kinase C (PKC)eta-mediated PKC mu activation modulates ERK and JNK signal pathways J Biol Chem 2002 277 8 6490 6496 10.1074/jbc.M106083200 11741879

[B59] Tan M Xu X Ohba M Ogawa W Cui MZ Thrombin rapidly induces protein kinase D phosphorylation, and protein kinase C delta mediates the activation J Biol Chem 2003 278 5 2824 2828 10.1074/jbc.M211523200 12431976

[B60] Valverde AM Sinnett-Smith J Van Lint J Rozengurt E Molecular cloning and characterization of protein kinase D: a target for diacylglycerol and phorbol esters with a distinctive catalytic domain Proc Natl Acad Sci USA 1994 91 18 8572 8576 10.1073/pnas.91.18.8572 8078925 PMC44648

[B61] Endo K Oki E Biedermann V Kojima H Yoshida K Johannes FJ Kufe D Datta R Proteolytic cleavage and activation of protein kinase C [micro] by caspase-3 in the apoptotic response of cells to 1-beta -D-arabinofuranosylcytosine and other genotoxic agents J Biol Chem 2000 275 24 18476 18481 10.1074/jbc.M002266200 10764790

[B62] Kennett SB Roberts JD Olden K Requirement of protein kinase C micro activation and calpain-mediated proteolysis for arachidonic acid-stimulated adhesion of MDA-MB-435 human mammary carcinoma cells to collagen type IV J Biol Chem 2004 279 5 3300 3307 14607845 10.1074/jbc.M305734200

[B63] Vantus T Vertommen D Saelens X Rykx A De Kimpe L Vancauwenbergh S Mikhalap S Waelkens E Keri G Seufferlein T Doxorubicin-induced activation of protein kinase D1 through caspase-mediated proteolytic cleavage: identification of two cleavage sites by microsequencing Cell Signal 2004 16 6 703 709 10.1016/j.cellsig.2003.11.009 15093611

[B64] Nishikawa K Toker A Johannes FJ Songyang Z Cantley LC Determination of the specific substrate sequence motifs of protein kinase C isozymes J Biol Chem 1997 272 2 952 960 10.1074/jbc.272.2.952 8995387

[B65] Matthews SA Rozengurt E Cantrell D Characterization of serine 916 as an in vivo autophosphorylation site for protein kinase D/Protein kinase Cmu J Biol Chem 1999 274 37 26543 26549 10.1074/jbc.274.37.26543 10473617

[B66] Celil AB Campbell PG BMP-2 and insulin-like growth factor-I mediate Osterix (Osx) expression in human mesenchymal stem cells via the MAPK and protein kinase D signaling pathways J Biol Chem 2005 280 36 31353 31359 10.1074/jbc.M503845200 16000303

[B67] Vega RB Harrison BC Meadows E Roberts CR Papst PJ Olson EN McKinsey TA Protein kinases C and D mediate agonist-dependent cardiac hypertrophy through nuclear export of histone deacetylase 5 Mol Cell Biol 2004 24 19 8374 8385 10.1128/MCB.24.19.8374-8385.2004 15367659 PMC516754

[B68] Sun F Kanthasamy A Song C Yang Y Anantharam V Kanthasamy AG Proteasome inhibitor-induced apoptosis is mediated by positive feedback amplification of PKCδelta proteolytic activation and mitochondrial translocation J Cell Mol Med 2008 12 6A 2467 2481 10.1111/j.1582-4934.2008.00293.x 18298651 PMC2957660

[B69] Zhang D Kanthasamy A Yang Y Anantharam V Protein kinase C delta negatively regulates tyrosine hydroxylase activity and dopamine synthesis by enhancing protein phosphatase-2A activity in dopaminergic neurons J Neurosci 2007 27 20 5349 5362 10.1523/JNEUROSCI.4107-06.2007 17507557 PMC3407040

[B70] Kitazawa M Anantharam V Kanthasamy A Kanthasamy AG Dieldrin promotes proteolytic cleavage of poly(ADP-ribose) polymerase and apoptosis in dopaminergic cells: protective effect of mitochondrial anti-apoptotic protein Bcl-2 Neurotoxicology 2004 25 4 589 598 10.1016/j.neuro.2003.09.014 15183012

[B71] Afeseh Ngwa H Kanthasamy A Anantharam V Song C Witte T Houk R Kanthasamy AG Vanadium induces dopaminergic neurotoxicity via protein kinase Cdelta dependent oxidative signaling mechanisms: relevance to etiopathogenesis of Parkinson's disease Toxicol Appl Pharmacol 2009 240 2 273 285 10.1016/j.taap.2009.07.025 19646462 PMC2753722

[B72] Song C Kanthasamy A Anantharam V Sun F Kanthasamy AG Environmental neurotoxic pesticide increases histone acetylation to promote apoptosis in dopaminergic neuronal cells: relevance to epigenetic mechanisms of neurodegeneration Mol Pharmacol 77 4 621 632 10.1124/mol.109.062174 PMC2847769 20097775

[B73] Latchoumycandane C Anantharam V Kitazawa M Yang Y Kanthasamy A Kanthasamy AG Protein kinase Cdelta is a key downstream mediator of manganese-induced apoptosis in dopaminergic neuronal cells J Pharmacol Exp Ther 2005 313 1 46 55 15608081 10.1124/jpet.104.078469

[B74] Storz P Toker A Protein kinase D mediates a stress-induced NF-kappaB activation and survival pathway Embo J 2003 22 1 109 120 10.1093/emboj/cdg009 12505989 PMC140053

