# Supplementary figures and images for: Protein kinase D1 (PKD1) activation mediates a compensatory protective response during early stages of oxidative stress-induced neuronal degeneration
Source: Mol Neurodegener. 2011 Jun 22;6:43. doi: 10.1186/1750-1326-6-43 (PMC3145571; doi:10.1186/1750-1326-6-43)

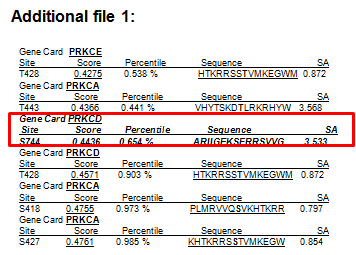

Supplement: Additional file 1 — Rat PKD1 amino acid sequence obtained from Swiss-Prot database (ID: Q9WTQ1) was analyzed using Scansite Motif software to identify the upstream PKCs that phosphorylate the PKD1 activation loop serine residues. The analysis done at high stringency shows that only PKCδ phosphorylates PKD1 at the activation loop residue Serine 744 site. [file 1750-1326-6-43-S1.TIFF]

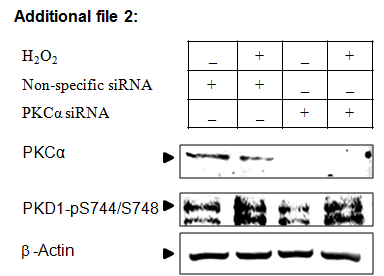

Supplement: Additional file 2 — N27 cells were transfected with 1 μM PKCα siRNA and non-specific siRNA and monitored for PKCα protein expression and PKD1pS744/S748 after H2O2 treatment. PKCα knockdown did not cause attenuation in PKD1 activation loop phosphorylation. [file 1750-1326-6-43-S2.TIFF]
